# *Capsicum* Waste as a Sustainable Source of Capsaicinoids for Metabolic Diseases

**DOI:** 10.3390/foods12040907

**Published:** 2023-02-20

**Authors:** Mursleen Yasin, Li Li, Michelle Donovan-Mak, Zhong-Hua Chen, Sunil K. Panchal

**Affiliations:** School of Science, Western Sydney University, Richmond, NSW 2753, Australia

**Keywords:** capsaicin, dihydrocapsaicin, metabolic disease, *Capsicum annum*, *Capsicum chinense*, extraction

## Abstract

Capsaicinoids are pungent alkaloid compounds enriched with antioxidants, anti-microbial, anti-inflammatory, analgesics, anti-carcinogenic, anti-obesity and anti-diabetic properties. These compounds are primarily synthesised in the placenta of the fruit and then transported to other vegetative parts. Different varieties of capsicum and chillies contain different capsaicinoid concentrations. As capsicums and chillies are grown extensively throughout the world, their agricultural and horticultural production leads to significant amount of waste generation, in the form of fruits and plant biomass. Fruit wastes (placenta, seeds and unused fruits) and plant biowaste (stems and leaves) can serve as sources of capsaicinoids which can provide opportunities to extract these compounds for development of nutraceutical products using conventional or advanced extraction techniques. Capsaicin and dihydrocapsaicin are two most abundantly found pungent compounds. Considering the health benefits of capsaicinoids, these compounds can help in reducing metabolic disease complications. The development of an advanced encapsulation therapy of safe and clinically effective oral capsaicinoid/capsaicin formulation seem to require evaluation of strategies to address challenges related to the dosage, limited half-life and bioavailability, adverse effects and pungency, and the impacts of other ligands antagonising the major capsaicinoid receptor.

## 1. Introduction

Food waste is a global issue, as almost one third of food produced is lost before it is consumed [1]. Simultaneously, the water, energy, land and fuel resources used along the food supply chain are also wasted during this process. When discarded into landfill, both the plant biowaste and food waste impact the environment by the emission of greenhouse gases [2]. Global increases in population and economic status have resulted in an increased demand for high-quality food. Megatrends are also quickly evolving for food consumption, which are influencing the development of local and global food industries [3]. There is an increasing demand for premium ready-to-eat, dried, frozen or canned foods ultimately resulting in more food waste from the food processing industry. In 2018, global food waste was reported to be 1.6 billion tonnes, causing a loss of USD 1 trillion economically [4], USD 900 billion socially and USD 700 billion environmentally [5]. Fresh fruits and vegetables make up a large proportion of food wastage [6]. The majority of fruit and vegetable losses in the developing countries occur at the harvesting and processing stages with smaller proportions lost at the consumption stage [5]. However, in industrialised countries, these losses occur mainly at the harvesting, grading and consumption stages, and we can assume this trend to become more widespread as socioeconomic levels increase globally [5].

Fruits and vegetables are important food groups for healthy human diets. Consumption of sufficient amounts of fruits and vegetables is important as these food groups provide micronutrients (vitamins and minerals), dietary fibre and bioactive phytochemicals, such as polyphenols and carotenoids [7]. An increased understanding of the importance of the phytonutrients contained in fruits and vegetables for health benefits has led to the increased demand for these important food commodities, which in turn facilitates healthier populations and changing dietary preferences and demands [8]. However, where consumer demand precedes supply chain capabilities, this increased production of fruits and vegetables combined with improper handling methods and limited infrastructure have created situations of huge post-harvest losses and biowaste from these important food commodities [8,9]. Fruits and vegetables contribute only 20% to the total food purchased, but they are responsible for half of the food waste [10]. It is estimated that food losses are highest for fruits and vegetables and may reach up to 60% of the total produce [9]. Among these losses, approximately 30% of fruits and vegetables are lost during processing of whole commodities [9]. Annually, 1,748 million tonnes of fruits and vegetable waste is produced globally [11]. Fruit and vegetable wastes are produced at all stages of the supply chain from production, transportation, storage, distribution and consumption. Fruit and vegetable wastes are most commonly generated at wholesale markets, supermarkets and during agricultural activities [12]. Vegetable wastes account for approximately 300 million tonnes out of 900 million tonnes produced globally [5]. According to FAO, the developed regions of Europe, America and Oceania are the highest contributors of food waste, as 52% of vegetable supplies in these countries is not consumed, whereas comparatively, contributions by high-income countries in Asia is 21% and South Asia and South-Eastern Asia is 17%. Latin America and sub-Saharan areas waste approximately 17% of vegetables produced, compounding the SDG-2 “Zero Hunger” challenge in these regions where the supply is also limited [5]. Similarly in Australia, approximately 3.1 million tonnes of food, equivalent to AUD 20 billion annually is wasted by consumers. Other than that, 2.2 million tonnes are discarded by the commercial and industrial sectors during processing and packaging [13].

The enormous amount of food waste can have severe impacts on the environment, economy and food security [14]. Food waste dumped in landfills releases about 4.4 gigatonnes of greenhouse gases annually and ranks third highest in the list of greenhouse gas producers [15]. Impacts on greenhouse gas emissions, together with the limited availability of disposal sites and large costs on waste transportation, have shifted focus to treating waste more efficiently using green and sustainable processes. Repurposing food waste seems to be another option, which may help address food insecurity. In 2021, the Food and Agriculture Organisation of the United Nations (FAO) conceded that despite previous optimism, the world is not progressing towards achieving the UN’s Sustainable Development Goals (SDGs) 2.1 or 2.2 to ensure access to sufficient, safe and nutritious food or eradicate all forms of malnutrition [16]. Therefore, repurposing wasted fruits and vegetables will have a beneficial impact, not only on the environment, but also on food security and economic development [9].

Innovative technologies and techniques targeting the reuse of food waste may help reduce greenhouse gas emissions [17]. One such method is the exploitation of this waste to produce value-added products. Microorganisms can be used to metabolise organic molecules and convert them into useful products to be used as bioactive compounds, biopolymers, biofuel, functional foods, pharmaceutical preparations and in various other biotechnological applications [18]. These bioactive compounds include dietary fibre [19], nutrients, proteins, peptides, phenolic compounds, polysaccharides, proteins, flavours and phytochemicals [20]. These products are enriched with antioxidant, anti-microbial, cardioprotective and anti-cancer properties and are also helpful in colour stabilisation and prevention of food spoilage [21]. For most fruits and vegetables, the only consumable portion is the epithelium or pulp, with the rest discarded. The leftover parts, such as seeds, peduncle, peel, loculum and base are enriched with such bioactive and nutraceutical compounds [22]. *Capsicum* (*Capsicum annum* L.), or sweet pepper, is one such example of a vegetable that has a short shelf-life, especially in regions lacking cold-chain post-harvest storage [23]. *Capsicum* spp., including chilli varieties, are rich sources of bioactive and nutraceutical compounds, in particular, capsaicinoids. This review will summarise *Capsicum* spp. as a sustainable source of these compounds along with their health benefits, extractions methods and potential applications for human health. This concept of repurposing the unused food products for the development of another product will help in targeting the SDG to reduce the per capita food waste to half by 2030 at retail and consumer stages [24].

## 2. *Capsicum* spp. as Source of Capsaicinoids

*Capsicum* fruits (capsicums and chillies) are enriched with carbohydrates, proteins, calcium, magnesium, potassium and vitamins A, C and E. The unique compounds of the genus *Capsicum* are called capsaicinoids that cause the heat or pungency of these fruits [25]. Capsaicinoids have demonstrated many beneficial health properties for humans [26] and help plants in survival and seed dispersal [27]. These secondary metabolites can be extracted through several different techniques for the sustainable development of nutraceuticals and supplements [8]. Generally, capsaicinoids are obtained from fruits as these are produced in epidermal layers of fruit placenta [28]. However, these compounds can also be distributed across the different plant tissues and could also be extracted from other parts of the plants [29].

**Figure 1:**
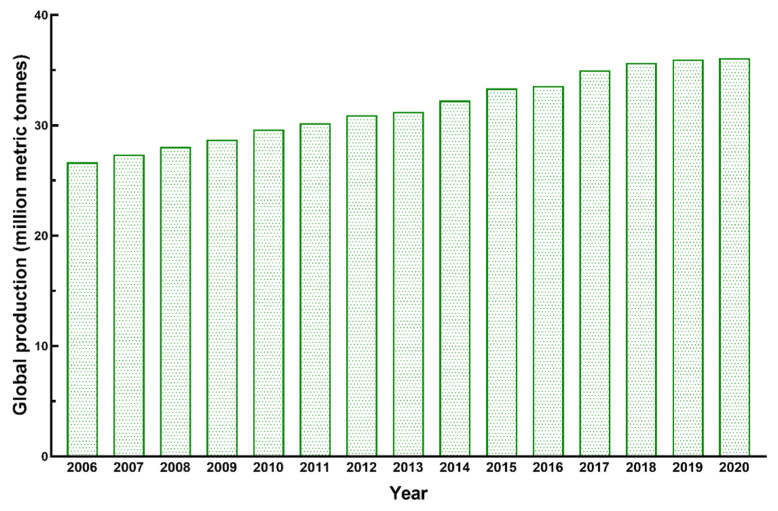
Global production of capsicums from 2006–2020.

The production of *Capsicum* spp. has increased globally by 35% (2006–2020) (Figure 1) [30,31] and is considered to be one of the most commercially cultivated vegetables [32]. However, almost 46% of capsicum crops is estimated to be wasted annually [33]. Approximately 56% of the capsicum plant is considered as non-saleable and may be treated as waste depending upon the requirement of the grower [10]. According to the recent data, Asia is the highest producer with approximately 65% of global *Capsicum* production. The share of the Americas, Europe, Africa and Oceania is 13.3%, 11.9%, 10.1% and 0.2%, respectively. According to the FAO [34], China was the top producer with almost 19 million tonnes of production in the year 2018–2019 [35].

The *Capsicum* genus belongs to the *Solanaceae* family originating in the tropical and humid parts of South America and Central Asia. Over 200 species of *Capsicum* exist, however, five are economically important and cultivated around the world. These are *C. annuum*, *C. frutescens*, *C. chinense*, *C. baccatum* and *C. pubescens* [36]. *Capsicum* spp. fruits are separated into edible and non-edible portions for consumption or industrial processing. Pericarp is the largest part of fruit which is used for consumption. The rest of the fruit parts i.e., seeds, placenta and peduncles are wasted. Pericarp was identified as the major portion of fruit for both *C. chinense* and *C. baccatum* species, i.e., 62.5% and 85.1%, respectively [37]. The discarded portion (seeds and placentas) enriched with capsaicinoids made almost 33% of the fruit in *C. chinense* and 14% in *C. baccatum*. Therefore, these parts could be recovered and processed for extraction of capsaicinoids to create valuable nutraceutical products. Moreover, the vegetative parts of plants, such as leaves and stems, are considered biowaste material in most cases. Unless the grower uses this biowaste as a stockfeed or a low-energy-conversion biofuel, the only way to use this waste is to process it as compost. However, reports suggested that these parts also contain valuable capsaicinoids [38,39]. Though synthesised and stored in placenta, some capsaicinoids travel to other tissues, such as leaves and stems [38,39]. Thus, these parts, so called plant and fruit wastes, can be processed for the extraction of their bioactive compounds.

In consumption terms, the reason for the popularity of *Capsicum* genus is the pungency and heat of their fruits, measured in Scoville heat units (SHU) [40]. The hottest chillies belong to *C. chinense* Jacq. Species, such as the Carolina Reaper (~1,500,000 SHU) and Ghost Chilli (~1,000,000 SHU). Chillies are mostly consumed for their spicy, burning or pungent ‘hot flavour’ in various forms, as fresh fruits, dry chilli flakes, powdered form, paprika oleoresin and chilli paste [41]. The pungent trait of this genus is due to the presence of capsaicinoids [42]. Compared to chillies, these compounds are also present in lower amounts in capsicum fruits. Capsaicinoids have been studied for their health benefits and many beneficial effects have been identified, including metabolic improvements [43,44,45,46,47,48,49]. Due to potential health benefits that may require a dosage unattainable through diet alone, capsaicinoids can be formulated as supplements against metabolic abnormalities in diabetes and obesity.

## 3. Capsaicinoids

### 3.1. Pungency

**Figure 2:**
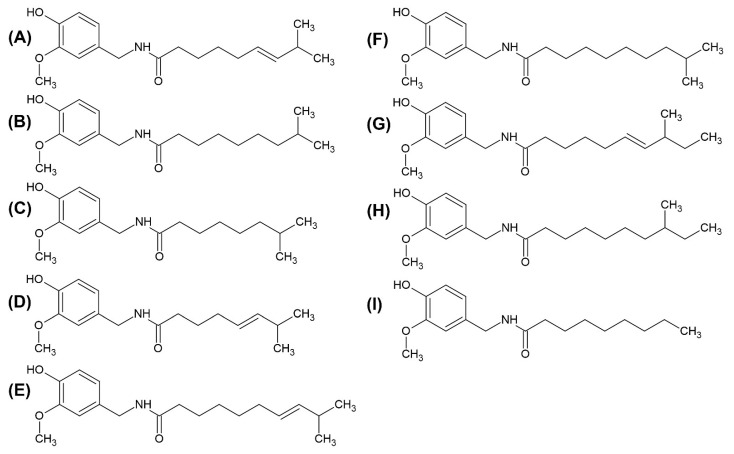
Structures of capsaicinoids: (**A**) capsaicin, (**B**) dihydrocapsaicin, (**C**) nordihydrocapsaicin, (**D**) norcapsaicin, (**E**) homocapsaicin I, (**F**) homodihydrocapsaicin I, (**G**) homocapsaicin II, (**H**) homodihydrocapsaicin II and (**I**) nonivamide.

The pungency or the burning sensation of fruits from the *Capsicum* genus is due to the accumulation of secondary metabolites called capsaicinoids. These are group of non-volatile alkaloids synthesised via a complex chain of chemical reactions [50]. In these reactions, phenylpropanoids and branched-chain fatty acids are converted into capsaicinoids [50]. The pungency of capsaicinoids can be linked to the amide bond between vanillyl ring and an acyl chain [51]. The two major capsaicinoids are capsaicin (8-methyl-*N*-vanillyl-*trans*-6-nonenamide; Figure 2A) and dihydrocapsaicin (8-methyl-*N*-vanillylnonanamide; Figure 2B); these two capsaicinoids contribute almost 90% of the pungency [52,53]. The other capsaicinoids (Figure 2C–I) are nordihydrocapsaicin, norcapsaicin, homocapsaicin I, homodihydrocapsaicin I, homocapsaicin II, homodihydrocapsaicin II and nonivamide [54]. *C. chinense* has the highest number of pungent varieties among capsicum species [55]. In these fruits, 62% of capsaicinoids are found in the placenta, 37% in seeds and only 1% in the pericarp [56]. The pungency of the fruit increases with the ripening and reaches the peak stage after 40 to 60 days of pollination, after which it declines rapidly due to peroxidases enzyme activity [57]. Capsaicinoids are genetic and genotypic dependent compounds which are also affected by crop and post-harvest management, maturation stage of the fruit, as well as environmental factors, such as temperature, light, water stress and soil fertility [58].

### 3.2. Health Benefits from Capsaicinoids

In the fields of pharmacology, nutrition, chemical weapons and shark repellents, there are almost 1000 registered patented products made of capsaicinoids, their synthetic analogues and oleoresin (the viscous extract) [38]. Capsaicinoids are used for treating several inflammatory conditions due to their physiological, pharmacological and anti-microbial effects [59]. This group of compounds also has many promising effects on the gastrointestinal tract, respiratory, cardiovascular, sensory and thermoregulatory systems [60,61].

**Figure 3:**
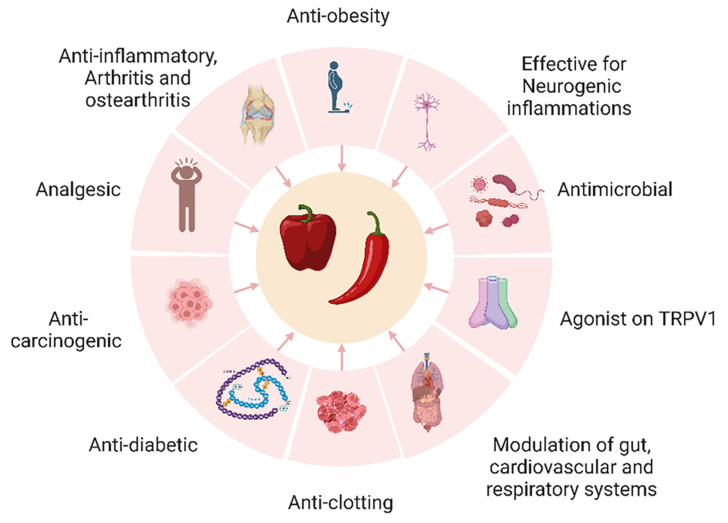
Health benefits of capsaicin.

**Figure 4:**
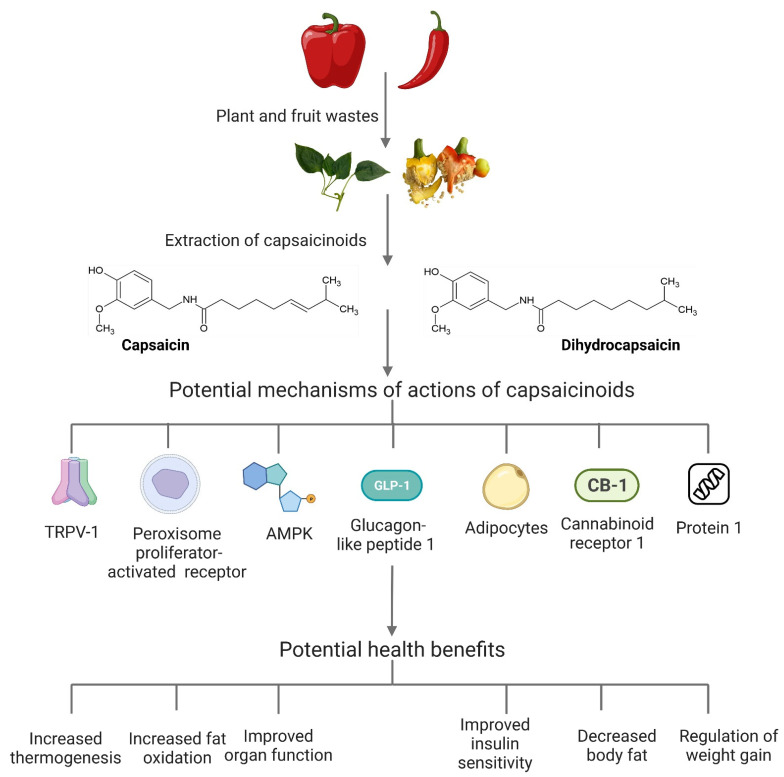
Potential mechanisms of health benefits of capsaicinoids that can be extracted from capsicum and chilli wastes.

Figure 3 summarises some of the benefits of capsaicin. Capsaicin is reported to be effective for arthritis [62], osteoarthritis [63] and neurogenic inflammations, such as the burning and stinging of hands, mouth and eyes [64]. It also has anti-cancer [65], anti-bacterial [66], anti-virulence [67], analgesic [68], anti-diabetic [69] and anti-inflammatory properties [70]. Capsaicin is an agonist on transient receptor potential vanilloid channel 1 (TRPV1) receptors present in many metabolically active tissues [43,71]. Previous studies have confirmed the effectiveness of low doses of capsaicin in improving metabolic disorders [43,49,71,72]. These effects of capsaicin and capsaicinoids, mediated through selective actions on TRPV1, can modulate the browning of adipocytes, activation of AMP-activated protein kinase, peroxisome proliferator-activated receptor, uncoupling protein 1 and glucagon-like peptide 1. Modulation of these pathways by capsaicin can increase thermogenesis and fat oxidation, improve insulin sensitivity, decrease body fat and improve organ functions (Figure 4) [43,61,71]. Suppressed upregulation of the cannabinoid receptor 1 by capsaicin mediated by butyrate producing gut bacteria also appears to contribute to the regulation of weight gain and insulin resistance [73].

The medicinal effect of capsaicinoids on the human body is dependent upon their dose and exposure time [74]. However, exposure to higher dosages, of more than 100 mg/kg body weight, for prolonged periods of time can cause ulcers, irritation and cancers of the prostate, liver, stomach and duodenum [75]. Therefore, it is important to monitor the daily dose of capsaicin and other pungent compounds, a necessity that can be facilitated by supplementation rather than fresh ingestion. Research showed that 8% capsaicin patch can be used to treat chemotherapy-induced neuropathy symptoms [76]. It has also been observed to provide relief against HIV-associated neuropathic pain, post-herpetic neuralgia [77] and cluster headaches [77,78]. Capsaicinoids have been widely researched and used for analgesic purposes [79]. They can be used in low dosages orally or in the form of local administration (creams, ointments, patches, etc.) for treating inflammation and pain from rheumatoid arthritis, fibromyalgia and chemical hyperalgesia [80]. The less concentrated analgesic creams and patches are available as over the counter medicine with 0.025–1% capsaicin [81]. A recent review has extensively described the use of capsaicin in neuropathic pain treatment without altering the large motor nerve fibres [82]. Moreover, these compounds also have anti-clotting properties and minimised platelet aggregation and activity of clotting factors VIII and IX [83], although this property is not dependent on TRPV1 channels [84].

Capsaicin has been reported for its antimicrobial potential against gram-positive and gram-negative bacteria, for example, *Bacillus subtilis*, *Streptococcus mutans*, *Streptococcus pyogenes*, *Staphylococcus aureus* and *Escherichia coli* [85]. Habanero, serrano and morron chilli extracts inhibited the growth of *Listeria monocytogenes*, *Bacillus cereus*, *Staphylococcus aureus* and *Salmonella enterica* [59]. Jalapeño peppers were also found to be effective against *Listeria monocytogenes* [86]. Moreover, capsaicin also has antifungal properties against *Penicillium expansum*, *Trametes versicolor* and *Gleophyllum trabeum* [85]. Capsaicinoids have shown potential to treat some viral diseases, such as herpes simples virus in guinea pigs [67]. Capsaicinoids supplements are also available for weight loss management; the recommended serving is <10 mg capsaicin/day or <10 mg dihydrocapsiate/serving. There are safety concerns regarding higher usage of hot peppers or capsaicinoids supplement at 33 mg/day for 4 weeks or 12 mg/day for 12 weeks [87].

Further pre-clinical and clinical benefits have been highlighted in Table 1 andTable 2. With all these health benefits, capsaicinoids hold an important place in the field of nutraceuticals and pharmaceuticals. In order to develop capsaicinoid-based products that can provide health benefits, purified or concentrated forms of capsaicinoids are required in larger quantities that can be produced in a highly cost-efficient manner. Fruit and plant biowastes of capsicum and chilli may provide a suitable option for extracting capsaicinoids in concentrated form.

### 3.3. Capsaicinoids from Capsicum and Chilli Waste

The reproductive parts of the plant, such as the epidermal layer of the fruit placenta (the non-edible portion), is responsible for the major production and storage of capsaicinoids [38]. The discarded placenta contains the highest levels of capsaicinoids, i.e., 79% and 51% of the total capsaicinoid content in *C. chinense* and *C. baccatum*, respectively. When the discarded seeds are also considered, these non-edible wastes account for 86% and 77%, respectively [37], which enters landfill rather than the food-chain. Capsaicinoids are also present in the edible pericarp of capsicum fruits. Smaller amounts can also be found in other vegetative parts of the plant, such as leaves and stem [38] which are produced in larger volumes than the harvested fruits and are primarily considered biowaste. In Jalapeño pepper, the highest capsaicin and dihydrocapsaicin concentrations were observed in the placenta with only 0.7% additionally found in other vegetative organs, while in Padrón pepper, higher capsaicinoids were observed in the leaves and stems, as compared to Jalapeño pepper [101]. A study on Padrón pepper suggested that capsaicinoids concentration was higher in the leaves and apical portion of the plant, as compared to the stem and basal segments [39].

One study investigated the amount of capsaicinoids collectively from the calyx and peduncle of *C. annuum* fruits, the parts which are discarded as waste material. Four different genotypes were subjected to analysis, i.e., red chilli pepper, chilli Samandağ pepper, red sweet pepper and green hot Apraş pepper [102]. The highest concentrations of capsaicinoids were reported in chilli Samandağ pepper followed by red chilli pepper, i.e., 154.39 µg/g and 102.73, respectively. The lowest amounts of these pungent compounds were unsurprisingly observed in red sweet pepper, i.e., 0.64 µg/g [102].

Capsaicinoids were extracted from seeds, pericarp and placenta from 46 samples of *C. chinense* using ultrasound-assisted extraction. It was observed that the seeds had more capsaicinoids, as compared to the pericarps, ranging between 0 to 2270 mg/kg of fresh weight [103]. It can be due to the close proximity or direct attachment of the seeds with the placenta [104]. However, it is to be noted that capsaicinoid concentrations depend on the *Capsicum* variety and the portion of placenta that remains attached to the seeds [41,103].

To our knowledge there has not been a thorough investigation into the influence of growing techniques, environmental stressors, or plant-nutrient influences on capsaicinoids, although some studies have included some of these metabolites in their analysis of fruits and seeds [58,105]. Reductions in plant-nutrients by 20% and 40% for the week prior to harvest increased capsaicin levels in fruits by 16% and 18%, respectively [106]. However, when the nutrient was reduced by 60% and 80%, capsaicin decreased by 22% and 43%, in comparison to the control, respectively. When grown under increased nitrogen concentrations (additions of 153 and 230 kg N/ha), significant increases in both capsaicinoids were seen, however a generous supply of >300 kg N/ha resulted in a significant reduction in most years [107]. Interestingly, Das et al. [108] chose to assess an Indian landrace chilli (fruit) grown under different soil-types, organic and inorganic fertiliser regimes, and geographical regions. This study concluded that each factor; soil-type, fertiliser and even year replicated (and therefore climate variations) can have an influence over increasing capsaicin when compared to the control. These and other studies infer that both plant nutrition and environmental stress may influence capsaicinoids production in edible parts, however plant biowaste, for the most part, is absent. To address the United Nations’ SDG 12: Ensuring sustainable consumption and production patterns, we need to reconsider how all waste streams, including agricultural biowaste, can be utilised for maximum output and minimum waste.

In Mexico, annual production of chilli is 3.2 million tons and it is the major export product of the country [109]. To meet the quality standards, a large amount of the crop is discarded, which is 18.4% of the total national crop. The discarded portion is mostly seeds, immature, incomplete and defective fruits. These fruit parts have the potential to be valorised [110].

The agro-waste from *Capsicum* crops can be utilised to make nanoparticles. A study used the extracts from the leaf, stem and roots of *C. chinense* to prepare gold and silver nanoparticles. They used the extracts from waste, as a reducing or stabilising agent aiming for the green synthesis of nanoparticles [111]. Another way to reduce waste impact of capsicum agro-waste is the extraction of the *C. annum* stem fibre. The fibre is then proposed to be used as an alternative raw material in automotive applications as interior panels and dashboards [112].

## 4. Extraction of Capsaicinoids

Capsaicinoids can be extracted from capsicum and chilli fruits and wastes through several different methods. One of the restrictions for the extraction process is that these compounds are soluble in organic solvents. The extraction depends on their solubility in the used solvent and it is regulated by temperature and solvent polarity [113]. Thus, it is essential to develop a suitable technique for the extraction process which does not affect the quality of capsaicinoids [114]. These compounds are sensitive to varying environmental conditions, so it is necessary to evaluate these factors during extraction, processing, commercialisation and consumption [115]. The extraction methods can be divided into conventional and modern techniques. Some of the techniques that are usually followed for capsaicinoids extraction are described here.

### 4.1. Conventional Extraction Techniques

These methods are based upon extraction efficiency of different solvents, heat application or different time durations for extraction. Although cheaper to run, these methods may not be safe (depending on the solvents used) and quality efficient. They require longer extraction times, larger volumes of solvents, limited range for extraction selectivity and cause thermal degradation of the extracts [116].

#### 4.1.1. Conventional Solvent Extraction

This is the most used technique for capsaicinoids extraction. The process is based on several operating parameters, such as the type and concentration of solvent, temperature, time, solute to solvent ratio and number of extraction steps. Methanol, ethanol, acetone, acetonitrile and toluene are some of the commonly used solvents. Many researchers have followed this process for capsaicinoids extraction from different capsicum and chilli varieties. Methanol has been used as a common solvent for fresh and dried Habanero chilli pepper and it was observed to be an effective extraction method [117]. Another study used acetone for extraction from Serrano and Tabasco peppers [118]. Ethanol and acetone extraction methods are more affordable and efficient for extraction of total capsaicinoids from the placenta of Habanero chilli pepper [119]. Further, ethanol and acetonitrile gave the best extraction results from fresh chilli powder while acetone was a better solvent for dried pepper [120]. This may be due to the presence of water in chilli fruits which affects the polarity of solvents during the extraction process [120]. During comparison, acetonitrile was a better solvent than acetone [121]; further, extracts of the former were more pungent than the latter one [121]. Based on the operating conditions, solvent extraction results in a high yield of capsaicinoids. However, the disadvantage of this conventional technique is that it requires higher extraction costs due to the longer time and large solvent volume [122].

#### 4.1.2. Soxhlet Extraction

Soxhlet extraction is a simple and effective solvent extraction method. It has been used for a wide range of samples, such as soils, sediments and animal and plant tissues. This method has been used for comparison of capsaicinoids extraction yield with the modern techniques [123]. This method provides a better interaction of solute with solvents which ultimately solubilises more compounds in the extraction material to give a higher yield of the desired compound. This is because the solvent in this method is used at its boiling temperature [123]. It has been used for the assessment of capsaicinoids and phenolics from *C. baccatum* L. with the use of hexane, ethyl acetate, ethanol and methanol solvents. Hexane and ethyl acetate provided increased capsaicinoid yields, as compared to ethanol and methanol [42]. The Soxhlet technique is also being used for Habanero chilli pepper [122] and Biquinho pepper [124]. The highest capsaicinoid yields were obtained from this method, as it requires a longer extraction time, higher temperature and solvent ratio, which makes them more readily soluble [124]. A study reported the recovery rate of capsaicinoids up to 92% with the Soxhlet method and ethanol as solvent. They also compared Soxhlet with maceration and ultrasound-assisted technique. The recovery rate with Soxhlet was 5% greater than with the ultrasound-assisted technique [125].

### 4.2. Advanced Extraction Techniques

The modern techniques are preferred over conventional ones because they are eco-friendly, require lower amounts of solvents and yield a higher content of extracted material [126]. Sustainable alternatives are being tested to replace conventional molecular solvents by ionic liquids. These are termed as green alternatives due to their low toxicity and biodegradability. Some of the advanced techniques for extraction include ultrasound-assisted, microwave-assisted, pulsed-electric field, supercritical fluid extraction, enzyme-assisted extraction and pressurised liquid extraction. Some of these techniques are discussed below.

#### 4.2.1. Ultrasound-Assisted Extraction

Ultrasound-assisted extraction is an environmentally friendly technology which involves the use of ultrasound waves to rupture the cell wall and breakdown sample pieces into smaller sizes [127]. The cell wall is ruptured by applying pressure in waves through the sample medium as compression and expression cycles [128]. The factors which are important to develop an optimised extraction protocol are ultrasound power, frequency, wavelength and time [128]. It requires a smaller amount of solvent, such as methanol and ethanol, short extraction periods and lower temperatures producing higher yields than conventional methods. Moreover, ultrasound waves do not affect the chemical and biological qualities of extracted compounds. The method requires less equipment investment and easy implementation that makes it accessible to be used by local industries [125]. Ultrasound-assisted extraction enhanced the capsaicinoids extraction from *C. baccatum* L. by 26%, as compared to conventional methods [42]. The extraction efficiency of capsaicin and dihydrocapsaicin was 80% from Habanero chilli pepper via the ultrasound-assisted extraction method using ethanol and water as solvents [129]. This method was used to extract capsaicinoids from *C. chinense* pericarp, seeds and placenta using different solvents, such as toluene, acetone, isopropanol, *n*-hexane and methanol. The study proposed methanol as the best solvent to use with ultrasound-assisted extraction [103].

#### 4.2.2. Microwave-Assisted Extraction

The technique is a combined use of old solvent extraction and microwave radiations. The microwave energy heats the solution containing sample, stimulating the breakdown of cells which refines the porosity of the biological material and hence enhances the extraction process [130]. During the microwave process, dipole rotation of molecules take place, which results in the disruption of hydrogen bonds and movement of ions [130]. The application range of the microwave frequency is from 300 MHz to 300 GHz. The three factors that are considered for this protocol are solvent type, microwave power and extraction time [116]. During this method, cell integrity and shape are maintained as the method does not involve thermal degradation and oxidation. However, the disadvantage is that thermal stress and localised cell pressure can lead to faster cell rupture, as compared to other methods. The microwave-assisted extraction has many benefits, including reduced processing time, low solvent and energy requirements, simplicity and effectiveness, no secondary waste [131] and a uniform heating that results in simultaneous extraction [132]. The technique has not been used widely for capsaicinoids extraction except for the sample preparation of high-performance liquid chromatography analysis. The method was applied to *C. chinense* for capsaicinoids extraction using different ethanol and aqueous sodium salt solutions. The best solvent providing 85% extraction efficiency, was 20% ethanol and 25% NaH_2_PO_4_ aqueous solution [133], a result better than through conventional methods. Capsaicinoids were extracted using microwave technology from *Capsicum frutescens* with 99.5% ethanol as solvent. The yield obtained was 5.3 mg/g [134].

#### 4.2.3. Pressurised Liquid Extraction

The method of pressurised liquid extraction is the application of high pressure to keep the solvent in a liquid phase even after its boiling point [123]. It is also known by other terms as pressurised fluid extraction, accelerated fluid extraction, enhanced solvent extraction and high-pressure solvent extraction [135]. Pressurised liquid extraction requires almost 90% less solvent, as compared to maceration and Soxhlet procedures [126]. Temperature and high pressure are applied to accelerate the extraction process and solvent diffusivity into the matrix [126] and hence use less amount of solvent and short extraction time [123]. Automated equipment is used in this process, which has an inert atmosphere and no light, hence preventing oxidative degradation of extracted products. The technique was used to extract capsaicinoids from fresh Cayenne pepper, long and round marble peppers [136] and Habanero chilli peppers [137] which resulted in a high recovery of capsaicin. In a study, extraction of capsaicinoids from Cayenne pepper was performed with three solvents (ethanol, methanol and water) using pressurised liquid extraction technique. The highest concentration was obtained with methanol, i.e., 450 µmol/kg and the least with water [136]. The results suggested that pressurised liquid extraction produced higher extraction yields than the conventional techniques and it was suggested as a green alternative procedure because of its short extraction time [116].

## 5. Potential Applications of Capsaicinoids

With many health benefits, including those related to metabolic health, capsaicinoids have received accumulating attention in the development of therapeutics to treat or enhance treatment of metabolic disorders [43,49,71,72]. Skin, oral, parenteral and aerosol administration therapies have all been investigated, particularly in preclinical studies, for a multitude of therapeutic functions [138]. Oral therapies appear to be preferred over other choices in studies evaluating the metabolic benefits of capsaicinoids [49,72,73,138,139,140]. This preference aligns with the high spatial restriction of effect mediated by the TRPV1 receptors upon the epidermal or intradermal application of capsaicin [141]. The choice is also consistent with general consumer preferences for oral therapies over injections and inhalation [142,143]. The bioavailability and half-life of capsaicin are very low in the plasma and these are independent of the routes of administration [138]. It is well grounded to develop a drug delivery system for capsaicinoid/capsaicin to improve metabolic outcomes.

The challenges to formulate a clinically effective therapy using capsaicin or a combination of capsaicinoids are related to the physical, physiological and pharmacological characteristics of those compounds. The key challenges reported include the establishment of dosage (dose and exposure time), limited half-life and modest bioavailability [74,141,144]. Other aspects that may influence efficacy likely relate to the adverse effects [145,146,147] and the presence of competitor ligands for TRPV1 [148].

### 5.1. Dosage

Despite ample pre-clinical evidence, there is insufficient clinical evidence to help establish effective doses of capsaicinoids or capsaicin as therapeutic options targeting metabolic disorders [74,149]. The limited dietary capsaicinoids/capsaicin intake data indicates a typical average daily intake of approximately 1 to 240 mg capsaicinoids (i.e., 0.01 mg/kg body weight to 4 mg/kg body weight), depending on habitual dietary preferences for capsaicinoids-containing foods [74]. This implies that for people with no habitual intakes of foods rich in capsaicinoids (e.g., a typical ‘Western’ diet), dietary intakes alone would unlikely meet the typical daily therapeutic dose of a few milligrams, as adopted in clinical trials with positive outcomes using commercial supplements [139,140]. The gap between consumption and therapeutic requirement is even markedly larger when this typical dietary intake estimate is compared with a dose of a couple of orders of magnitude higher administered in in vitro studies and animal models [72,73,144]. It is thus plausible that therapeutic formulations are needed to achieve the desired clinical outcomes in the treatment of metabolic disorders, at least for those with no habitual intakes of foods high in capsaicinoids. It is noteworthy that many doses in animal models and in vitro studies can be unsafe in humans [144]. In addition to irritation and ulceration, epidemiological data from populations with a tradition of high consumption of capsaicinoid-rich foods indicate that average daily capsaicin consumption of 90–250 mg (approximately a maximum of 4.2 mg/kg body weight/day) appears to augment the incidence of certain digestive system cancers, although there is no established causation [74]. Extremely high doses have also been demonstrated to be lethal in animal models [144]. More investigation on the therapeutic and safe doses in humans is warranted.

### 5.2. Bioavailability and Plasma Half-Life

The development of therapeutic capsaicinoid/capsaicin formulations also needs to consider the limited bioavailability and short plasma half-life [74,149]; both seem to be related to the fast passive absorption process in the upper digestive system secondary to the hydrophobicity of the molecules [149,150]. Once transported into the hepatic circulation, almost all of the compounds are transformed by the liver before entering the systemic circulation [144]. Further research is needed to elucidate whether it is the bioavailability of capsaicinoids/capsaicin or their metabolites that are crucial in modulating the downstream metabolic outcomes. This includes their influence on gut microbiome, which appears to contribute to metabolic regulation [49], generally considered to initiate mostly in the colon. It also awaits clarification whether the limited plasma half-life of capsaicinoids is critical in the delivery of the multitude of metabolic benefits. Time-dependent effects of capsaicin have been frequently reported [74]. However, a continued exposure to capsaicin at the TRPV1 receptor level may not be necessary, such as that happens to the TRPV1-expressed nociceptive nerve fibre [141]. Untangling the answers to the two aforementioned questions will likely influence the selection of delivery system(s) for the optimal delivery of capsaicinoids to the most relevant absorption, transformation and or action sites [149].

### 5.3. Delivery System

The selection of delivery systems for the development of a capsaicin formulation needs to consider materials that can counteract the adverse effects of capsaicin. The system needs to eliminate or mask the pungency that prevents many from consuming an adequate amount of capsaicinoid-rich foods [149]. It also needs to reduce the side effects, such as irritation, gastrointestinal cramps, stomach pain, nausea, diarrhoea, vomiting, erythema and pain at the site of application and help in improving compliance when taken as a nutraceutical or supplement [145,146,147].

One way would be to introduce encapsulation as a way of delivering capsaicin to its site of action and avoid pungency and contact with unwanted parts of the gut or gut environment [151]. There seems to be various emulsifiers and wall materials used alone or in combination for encapsulation. The main types of materials used, include sources of carbohydrates, proteins and water-soluble gums. For example, whey protein combined with octenyl succinic anhydride-modified starch has been used to improve the stability and solubility of capsaicin. Fenugreek dietary fibre and cellulose combined have been used to create a sustained release capsaicin supplement [139]. A comprehensive evaluation of capsaicinoid or capsaicin specific encapsulation systems is warranted to inform the formulation of such therapies based on the best available evidence. In addition to wall materials, oral delivery vehicles using various hydrophobicity-friendly microencapsulation materials and techniques, each with its own merits and limitations, are emerging [149]. An informative evaluation, specific for capsaicin and primarily of in vitro studies and in animal models has been published previously [149].

Another solution to the challenges of pungency is to consider non-pungent analogues, capsinoids, from the red pepper variety, CH-19 Sweet (*Capsicum annuum* L.) [145]. These analogues have been found to stimulate TRPV1 receptors along the gut in the same manner as capsaicin, but they either are not active in the oral cavity (e.g., capsin) or demonstrate a remarkably higher heat-sensation threshold [140,145]. They appear to exert modest metabolic effects similar to capsaicin.

### 5.4. Interactions

Compounds that may influence the therapeutic effect of the capsaicinoid/capsaicin formulation may need to be examined when preparing consumption recommendations. Key examples of such compounds include modest TRPV1 antagonists 25-OHD (active form of vitamin D) [148] and oleic acid (a long-chain monounsaturated fatty acid) [152]. Since both are common in diets, it may be important to advise consumers strategies to minimise overlap between intakes of foods or supplements high in those two compounds and intakes of the capsaicinoid/capsaicin therapy, if clinically meaningful antagonising effects are found. It may be equally important to evaluate the clinical impacts of capsaicinoid/capsaicin supplementation on absorption of carotenoids and its conversion to vitamin A. An increased absorption of β-carotene and lutein, but a decreased concentration of retinol in the presence of capsaicin in vivo has been reported [153]. It is unclear whether a similar effect would be seen in the assimilation of other fat-soluble vitamins. The bioavailability of a common statin therapy, pitavastatin, seems to be enhanced by long-term consumption of capsaicin in a dose-dependent manner in a rodent model [154]. Clear messages about strategies to mitigate possible interactions between capsaicinoid/capsaicin consumption and these aforementioned nutrients and drug therapies likely help maximise the desired therapeutic effects of the nutraceutical products while reducing risk of impacts on the absorption and metabolism of other dietary or medicinal components.

## 6. Conclusions and Future Perspectives

The increasing world population requiring an increased food supply is a major cause of increased food production and processing, leading to increased food waste generation. Careful consideration should be given before discarding any plant-based material into landfill as value-added products can be obtained from some of these wastes. One such waste is derived from the production of capsicums and chillies, which may be enriched in capsaicinoids. These compounds, with their health benefits, have potential in treating many metabolic disorders. Thus, their extraction with appropriate techniques will provide suitable material for product development. The on-farm waste (fruits and plants), from capsicum and chilli crops, is usually dumped or treated as compost. Therefore, future studies can focus to devise sustainable and cheaper extraction techniques to help farmers valorise the horticultural/agriculture waste. This will not only reduce the impact of horticultural waste on the environment, but also become a source of secondary income for the growers. Further, health benefits of these compounds will help in developing treatment strategies for metabolic disorders and hence help in reducing the prevalence of these metabolic disorders. Research effort is still required to address challenges related to the dosage, limited half-life and bioavailability, adverse effects and undesired heat sensation, and the impacts of other TRPV1 ligands. Strategies to minimise the influence of capsaicinoid/capsaicin on the absorption and metabolism of essential nutrients or bioactive compounds may also be required for the more holistic care of consumers.

## Figures and Tables

**Table 1:** Pre-clinical studies of capsaicinoids.

Study Model	Application	Results
Rat osteoarthritis synovium	Capsaicin injection	Reduced osteoarthritis phenotypes and M1 macrophage infiltration [88]
High-fat diet-induced obese mice	0.075% capsaicin	Decreased lipid accumulation in mesenteric and epididymal tissue [89]
Obese/diabetic KKAy mice	Dietary capsaicin	Reduced metabolic dysregulation [90]
Mice	Oral administration of capsaicin	Prevention of obesity in male wild-type mice [91]
Lewis rat	Capsaicin (for autoimmune neuropathies)	Reduced inflammation of the sciatic nerve [92]
Guinea pigs (high-fat diet)	Capsaicin (doses 2.5, 5, 10 mg/kg)	Reduce oxidative stress and endothelial dysfunction [93]
Human lung carcinoma cells	Erlotinib combined with 90% capsaicin (1:5 and 1:20)	Enhancement of cytotoxicity and inhibition of cell growth of erlotinib. Potential use as chemo-sanitiser for erlotinib [94]
Osteosarcoma cells	Capsaicin (100 µM) with cisplatin (16.7 µM)	Inhibitory effects on osteosarcoma cells, (apoptosis induction, cell cycle arrest and cell invasion inhibition) [95]

**Table 2:** Clinical studies of capsaicinoids.

Study Participants	Application	Results
Male/Female (18–56 years)	Capsaicinoids supplements (12 weeks)	Reduced appetite, improved body composition (waist: hip ratio) [96]
Women with gestational diabetes mellitus	Capsaicin supplements (4 weeks)	Improved postprandial hyperglycaemia and hyperinsulinemia, fasting lipid metabolic disorders [97]
Healthy Caucasian male/female	2.56 mg (1.03 g of red chili pepper) with meal	Negative energy balance, increased fat oxidation [98]
Male/Female (18–60 years)	135 mg capsaicin/day (3 months)	Increased fat oxidation during weight regain [99]
Healthy young men	150 mg capsaicin 1 h before exercise	Enhanced the activity of fat oxidation during low-intensity exercise [100]

## Data Availability

Not applicable.
